# Artificial light at night and risk of depression: a systematic review and meta-analysis

**DOI:** 10.1265/ehpm.24-00257

**Published:** 2024-12-26

**Authors:** Manman Chen, Yuankai Zhao, Qu Lu, Zichen Ye, Anying Bai, Zhilan Xie, Daqian Zhang, Yu Jiang

**Affiliations:** 1 School of Population Medicine and Public Health, Chinese Academy of Medical Sciences and Peking Union Medical College, Beijing, China; 2 School of Health Policy and Management, Chinese Academy of Medical Sciences and Peking Union Medical College, Beijing, China

**Keywords:** Outdoor artificial light at night, Indoor artificial light at night, Depression, Meta-analysis

## Abstract

**Background:**

Artificial light at night (ALAN) has been increasingly recognized as a potential environmental risk factor for mental health issues. However, no meta-analyses have been conducted to summarize the findings. This study aimed to evaluate the pooled associations between outdoor and indoor ALAN exposures and the risk of depression.

**Methods:**

Adhering to the PRISMA guideline, we conducted systematic searches across PubMed, Web of Science, EMBASE, Cochrane, and Ovid databases for studies published before May 1st, 2024.

**Results:**

A total of 7 studies (5 for outdoor ALAN and 2 for indoor ALAN) with a combined total of 560,219 participants were included in the meta-analysis. Specifically, a 1 nW/cm^2^/sr increase in outdoor ALAN was associated with a 0.43% (95% CI: 0.21%, 0.65%) increase in depression risk. Meanwhile, a 1 lux increase in indoor ALAN was associated with a 3.29% (95% CI: 0.85%, 5.79%) increase in depression risk. No potential heterogeneity was observed for outdoor ALAN exposure and indoor ALAN exposure. Subgroup analyses for outdoor ALAN indicated that development level, sample size, age group, sex, study design, modality of depression assessment, or adjustment of sleep-related variables in models may not be potential sources of heterogeneity. Sensitivity analyses confirmed the robustness of the findings, while evidence of publication bias was observed for studies on outdoor ALAN.

**Conclusions:**

Our findings suggest that both outdoor and indoor ALAN exposures are associated with increased risk of depression. These results underscore the importance of considering outdoor and indoor ALAN in public health strategies aimed at reducing depression risk. Nevertheless, further studies with prospective design are still warranted considering the limited study numbers.

**Supplementary information:**

The online version contains supplementary material available at https://doi.org/10.1265/ehpm.24-00257.

## Graphical Abstract

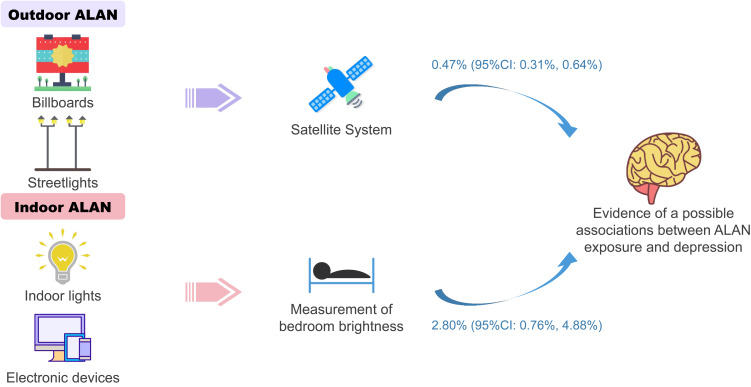

## 1. Introduction

In recent decades, artificial light at night (ALAN) has become a notable environmental health issue under the global background of urbanization [1, 2]. Although ALAN from various outdoor and indoor sources provided benefits on advancements and conveniences, growing evidence suggests that ALAN may have adverse health effects [1–4]. Epidemiological studies found that exposure to ALAN was associated with increased risks of cardiovascular diseases, metabolic disorders, neoplastic diseases (particularly breast cancer), and mental disorders, as well as mortality [5–13].

Depression is a prevalent mental health disorder with growing disease burden across the world. According to statistics from the global burden of disease (GBD) study, the prevalence of depressive disorders was 332.4 million, resulting in 56.3 million disability-adjusted life years (DALYs) in 2021 [14]. In recent years, various environmental factors have been linked to the development and exacerbation of depressive symptoms [15–18], and the potential role of ALAN has been receiving increasing attention [5, 6]. Evidence indicated that exposure to ALAN may result in disruption of circadian rhythms and sleep disturbance, which has been linked to increased risks of depression [6, 19–21].

However, epidemiological studies investigating the associations between ALAN exposure and depression have yielded mixed results [8, 9, 22–26]. For instance, several studies reported significant associations between ALAN and elevated risks of depression [22, 23], while others found no significant associations [25]. These discrepancies may be attributed to differences in epidemiological design, population characteristics, and indoor/outdoor settings [26]. Given the inconsistencies in the existing literature, a comprehensive synthesis of the current evidence would be beneficial for clarifying the associations.

To our knowledge, no meta-analyses have yet been conducted to systematically evaluate the pooled associations between ALAN exposure and depression. Related results would be beneficial for the development of public health strategies aimed at mitigating the adverse effects of ALAN on mental health. Accordingly, this systematic review and meta-analysis aimed to evaluate the pooled associations between outdoor and indoor ALAN exposures and the risk of depression.

## 2. Methods

Our study followed the Preferred Reporting Items for Systematic Reviews and Meta-Analyses (PRISMA) guidelines [27].

### 2.1. Search strategy and eligibility criteria

This meta-analysis conducted systematic searches across five literature databases, including PubMed, Web of Science, EMBASE, Cochrane, and Ovid, and identified related publications before May 1st, 2024, and studies identified in existing systematic reviews were also included, with no restrictions on region, publication type, or language. The keywords we used for identification were “light pollution, light exposure, artificial light, night*light, bedroom light, light at night, environmental light*, ambient light, domestic light, depression, depressive disorder, depressive symptom*, depressed mood”. The specific search strategy is available in Table S1.

The inclusion criteria were as follows: (1) the exposure variable was outdoor or indoor ALAN exposure; (2) the outcome variable was depressive symptoms assessed by a standard scale or diagnosed by physicians; (3) epidemiological study designs, including cross-sectional, case-control, cohort studies, and others; (4) reporting associations between ALAN and binary depression outcome (i.e., the presence or absence of depression) using quantitative measurements such as percent change (% change) in depression risk, odds ratio (OR) of depression, or other measures.

The exclusion criteria were: (1) not original population studies (e.g., case reports, reviews, or meta-analyses); (2) articles that did not report quantitative associations between outdoor or indoor ALAN and binary depression outcome; and (3) full-text access was unavailable.

### 2.2. Study selection and data extraction

Duplicate records were removed through EndNote version 21.0. Abstracts, titles, and full-texts of potentially eligible records were independently screening by two reviewers (M.C. and Y.Z.) to identify articles suitable for further review. Studies that had already been included in existing systematic reviews were also identified.

Data extraction was conducted independently by two investigators (M.C. and Y.Z.). Information extracted from all eligible studies included the title, authors, publication year, journal name, study location, duration, sample size, population characteristics (mean/median age, proportion of females), levels of outdoor or indoor artificial light at night (ALAN), measures of depressive symptoms, study design, effect estimates, 95% confidence intervals (CIs), and units of measurement for effect estimates. Any disagreements were resolved through consensus with a third investigator (Q. L.). All processes, including the management of original records, data extraction, and statistical analyses, underwent pre-submission review by two additional investigators (C.Y. and A.B.). During data extraction, results from all available models and exposure metrics were recorded, with preference given to results from the most adjusted model as reported in the primary text.

### 2.3. Quality assessment

The quality of all included studies was evaluated using the Newcastle-Ottawa Scale (NOS), a widely accepted tool for assessing the methodological quality of epidemiological studies across various designs, commonly used in systematic reviews and meta-analyses [28]. Additionally, for the cross-sectional studies included in this review, we utilized an adapted version of the NOS. Detailed criteria of the NOS tool can be found in Table S2. Quality assessment was conducted independently by two reviewers (M.C. and Y.Z.), with any discrepancies resolved through discussion with a third reviewer (Q.L.). The results of the quality assessments using the NOS are summarized in Table S3.

### 2.4. Statistical analysis

The Chi-square-based Cochrane Q statistic test and the I-squared statistic were used to assess between-study heterogeneity [27]. Due to potential heterogeneity among the included studies, standardized effect estimates were pooled using random-effects meta-analysis [11, 29]. Percent changes (%) in depression risk associated with ALAN were calculated across all eligible studies included in this meta-analysis [30, 31]. Effect estimates were standardized into percent changes with 95%CI, reflecting the risk of depression associated with a 1 nW/cm^2^/sr increase in outdoor ALAN, and a 1 lux increase in indoor ALAN exposure.

Subgroup analyses were conducted based on local development level (developed and developing regions), sample size (fewer than 20,000 and 20,000 or more), age (middle-aged and older versus younger), female proportion (less than 50% and 50% or more), study design (cross-sectional versus cohort studies), modality of depression assessment (physician-diagnosed records versus questionnaires), and adjustment of sleep-related variables in models (not adjusted versus adjusted). Differences between subgroups were assessed using standard error to test for significance in subgroup effect sizes. Additionally, meta-regression analyses were performed to explore potential modifications in effect due to population characteristics and study features, using the restricted maximum-likelihood estimator.

Sensitivity analysis was performed by serially excluding each study to determine the influence of individual studies on the overall results. Publication bias was assessed using funnel plots, Begg’s, and Egger’s tests [27]. All statistical analyses were conducted using R statistical software (version 4.3.0) with the packages “meta” and “forestplot.” Results with P < 0.05 (two-sided) were considered statistically significant.

## 3. Results

### 3.1. Study selection and characteristics

**Fig. 1:**
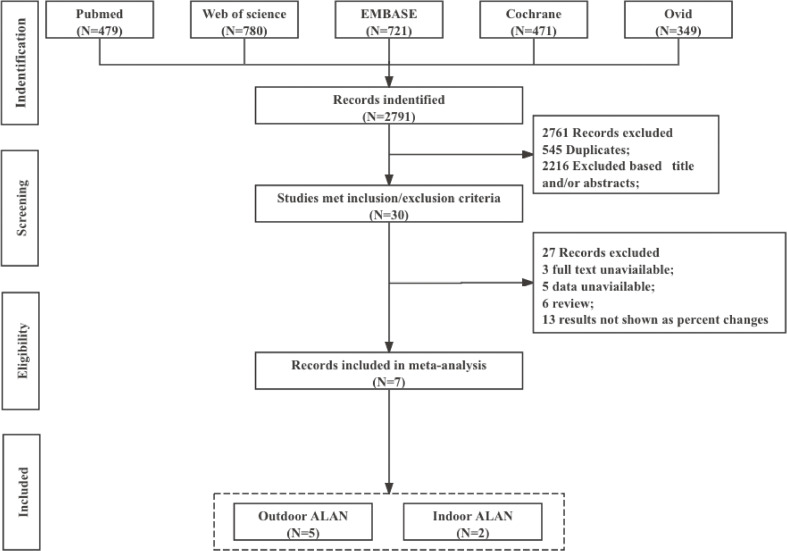
Flowchart of the literature search for the meta-analysis

The literature search and selection of eligible studies followed a comprehensive, pre-designed methodology, showed in Fig. 1. After the initial searching, 2791 records were identified from five databases. Following application of inclusion and exclusion criteria, 7 articles involving 560,219 participants were included in the meta-analysis [8, 9, 22–26]. Specifically, we identified 5 studies [9, 22, 23, 25, 26] on outdoor ALAN and 2 studies [8, 24] on indoor ALAN levels. All studies were assessed as having moderate or high quality. Detailed study characteristics are provided in Table 1.

**Table 1:** Characteristics of studies included in the meta-analysis

**Ref No**	**Article**	**Location**	**Study duration**	**Sample size**	**Mean/** **median age**	**Female** **(%)**	**Exposure ** **assessment**	**Outcome assessment**	**Confounders**	**Effect estimates**	**Design**	**Study quality**
**Outdoor ALAN**												
22	Jin (2023)	UK	2006–2010	298,283	56.58	53.4	NPP-VIIRS-like data	Physician-diagnosed records	Age, sex, ethnicity, education level, household income, diagnosed diabetes, diagnosed CVD/hypertension, Townsend deprivation, PM_2.5_, NO_2_, greenness percentage, and night-time noise	HR = 1.036 (1.004, 1.069) per 11.37 nW/cm^2^/sr increase	Cohort	High
23	Min (2018)	Korea	2009	220,258	40	54.3	National Centers for Environmental Information	CESD questionnaire	Age, gender, marital status, education, monthly income, job category, cigarette smoking, alcohol drinking, moderate physical activity, disease history (i.e., hypertension, diabetes, or hyperlipidemia), night noise, PM_10_, and parks and green spaces	OR = 1.29 (1.15, 1.46) per 47.25 nW/cm^2^/sr increase	Cross- sectional	High
25	Paksarian (2020)	USA	2019–2020	10,123	15.2	48.8	DMSP/OLS data	Modified version of the World Health Organization Composite International Diagnostic Interview	Age, sex, race/ethnicity, family income, parental, education, family structure, nativity, region, urbanicity, area-level population density, and socioeconomic status	OR = 1.07 (1.00, 1.15) per 1 nW/cm^2^/sr increase	Cross- sectional	High
9	Yu (2022)	China	2012–2018	21,036	50	51.35	NPP-VIIRS	CESD questionnaire	Age, drinking, education, gender, income, marital status, nationality, physical activity, obesity, residence, and smoking	OR = 1.052 (1.008, 1.097) per 1 nW/cm^2^/sr increase	Cohort	Moderate
26	Zhu (2023)	China	2009–2011	6,445	82.1	Unknown	DMSP/OLS data	CESD questionnaire	Age, years of education, family history of mental illness, physical activities, social activities, smoking, drinking, marital status, insomnia, PM_2.5_, and history of diabetes, hypertension, stroke, and region of each community	OR = 1.22 (1.06, 1.40) per 53.42 nW/cm^2^/sr increase	Cross- sectional	Moderate
**Indoor ALAN**												
24	Obayashi (2018)	Japan	2010–2014	863	72.5	51	Portable light meter placed facing the ceiling at the head of the participant’s bed	GDS questionnaire	Age, gender, body mass index, household income, hypertension, diabetes, sleep disturbances, bedtime, and duration in bed	HR = 1.72 (1.03, 2.89) per 12 lux	Cohort	High
8	Obayashi (2022)	Japan	2010–2019	2,947	69.3	60.6	Portable light meter placed facing the ceiling at the head of the participant’s bed	GDS questionnaire	Age, gender, smoking and drinking habit, income, education, physical activity, bedtime, duration in bed, daylength, and medication use	OR = 1.3 (0.98, 1.73) per 9.87 lux	Cross- sectional	High

Abbreviations: ALAN, artificial light at night; CESD, Center for Epidemiologic Studies Depression; DMSP/OLS, Defense Meteorological Satellite Program’s Operational Linescan System; GDS, Geriatric Depression Scale; NPP-VIIRS, the Suomi National Polar-orbiting Partnership-Visible Infrared Imaging Radiometer Suite; OR, odds ratio.

Note: Developed regions: Japan, Korea, UK, and USA; Developing regions: China; the study quality was assessed by the Newcastle-Ottawa Scale (NOS).

### 3.2. Associations of outdoor and indoor ALAN with depression

**Fig. 2:**
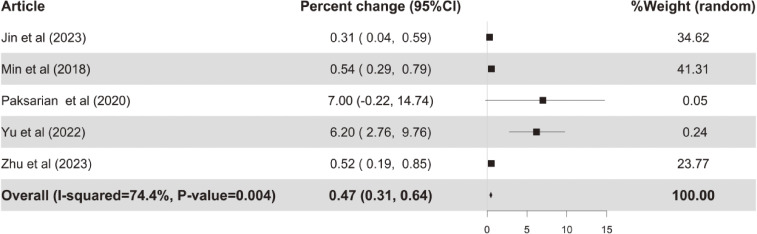
Forest plots of percent change in depression risk per 1 nW/cm^2^/sr increase in outdoor ALAN.

**Fig. 3:**
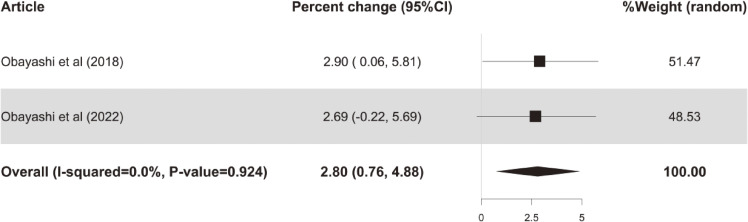
Forest plots of percent change in depression risk per 1 lux increase in indoor ALAN.

As shown in Figs. 2–3, the overall meta-analysis results indicated significant positive associations between outdoor or indoor ALAN exposure and depression. Specifically, a 1 nW/cm^2^/sr increase in outdoor ALAN was associated with a 0.43% (95% CI: 0.21%, 0.65%) increase in risk of depression, and a 1 lux increase in indoor ALAN was associated with a 3.29% (95% CI: 0.85%, 5.79%) increase in risk of depression. In addition, there was marginal but non-significant heterogeneity between the included studies for depression in association with outdoor ALAN exposure, with *P* for heterogeneity = 0.052. However, this phenomenon was not found in the association between depression and indoor ALAN exposure, with *P* for heterogeneity = 0.481.

### 3.3. Subgroup analysis and meta-regression analysis

Considering the relatively small number (n = 2) of studies on indoor ALAN, subgroup and meta-regression analysis were only conducted for outdoor ALAN studies. The results of subgroup analyses are shown in Table 2. Subgroup analyses suggested that development level, sample size, age group, sex, study design, modality of depression assessment, or adjustment of sleep-related variables in models may not be potential sources of heterogeneity (*P* for subgroup difference > 0.05) for studies on outdoor ALAN. Notably, only developed regions showed a significant association between outdoor ALAN and depression, while there was no significant difference in the effect estimates between developed and developing regions (*P* for subgroup difference > 0.05). Specifically, a 1 nW/cm^2^/sr increase in outdoor ALAN was associated with a 0.44% (95% CI: 0.21%, 0.67%) increase in depression risk in developed regions and a 2.25% (95% CI: −2.25%, 6.96%) increase in depression risk in developing regions. Results of meta-regression analyses were generally consistent with the above findings (Table 3).

**Table 2:** Subgroup analysis for the association between outdoor ALAN and depression

**Characteristic**	**Subgroup**	**Study ** **number**	**Estimate**	***P*-value**	**I-squared**	***P* for heterogeneity**	***P* for subgroup difference**
Development	Developed	3	0.44% (0.21%∼0.67%)	<0.001	56.30%	0.101	0.437
	Developing	2	2.25% (−2.25%∼6.96%)	0.332	78.80%	0.030	
Sample size	>20000	3	0.45% (0.21%∼0.68%)	<0.001	66.80%	0.049	0.484
	<20000	2	2.61% (−3.32%∼8.91%)	0.397	68.90%	0.073	
Age group	Middle-aged and older	3	0.37% (0.06%∼0.68%)	0.019	59.10%	0.087	0.442
	Younger population	2	2.67% (−3.08%∼8.76%)	0.371	67.20%	0.081	
Sex	Female proportion > 50%	3	0.45% (0.21%∼0.68%)	<0.001	66.80%	0.049	0.484
	Female proportion < 50%	2	2.61% (−3.32%∼8.91%)	0.397	68.90%	0.073	
Design	Cohort study	2	2.23% (−2.33%∼7.00%)	0.344	79.30%	0.028	0.457
	Cross-sectional study	3	0.47% (0.19%∼0.74%)	<0.001	49.20%	0.140	
Modality of depression assessment	Physician-diagnosed records	1	0.31% (0.04%∼0.59%)	0.027	—	—	0.343
	Questionnaires	4	0.47% (0.29%∼0.65%)	<0.001	64.60%	0.037	
Adjustment of sleep-related variables in models	Not adjusted	4	0.45% (0.26%∼0.64%)	<0.001	67.30%	0.027	0.641
	Adjusted	1	0.37% (0.11%∼0.63%)	0.005	—	—	

Note: Estimates were shown as pooled percent changes (%) and 95% confidence intervals (CIs) in risk of depression associated with 1 nW/cm^2^/sr increase in outdoor ALAN.

**Table 3:** Meta-regression analysis by potential modifiers for the association between outdoor ALAN and depression

**Group**	**Subgroup**	**Estimate**	***P*-value**
Development	Developed	Ref	0.761
	Developing	−0.05% (−0.37%∼0.27%)	
Sample size	<20000	Ref	0.700
	>20000	0.06% (−0.26%∼0.38%)	
Age group	Middle-aged and older	Ref	0.228
	Younger population	0.19% (−0.12%∼0.51%)	
Sex	Female proportion < 50%	Ref	0.700
	Female proportion > 50%	0.06% (−0.26%∼0.38%)	
Design	Cohort study	Ref	0.435
	Cross-sectional study	0.13% (−0.20%∼0.46%)	
Modality of depression assessment	Physician-diagnosed records	Ref	0.343
	Questionnaires	0.16% (−0.17%∼0.49%)	
Adjustment of sleep-related variables in models	Not adjusted	Ref	0.641
	Adjusted	−0.08% (−0.40%∼0.24%)	

Note: Estimates were shown as pooled percent changes (%) and 95% confidence intervals (CIs) in risk of depression associated with 1 nW/cm^2^/sr increase in outdoor ALAN.

### 3.4. Sensitivity analyses

Due to the limited number of studies focusing on indoor ALAN (n = 2), sensitivity analyses were only conducted for outdoor ALAN studies. Results from the sensitivity analyses, which excluded one study at a time, suggested that the associations between outdoor ALAN and depression were generally robust, as detailed in Table S4. Results of funnel plots, Funnel plots, Begg’s tests, and Egger’s tests are presented in Fig. S1 and Table S5, respectively. Begg’s tests showed no significant publication bias (*β* = 2.00, 95% CI: −6.00∼10.00, *P* = 0.624) among studies investigating the associations of outdoor ALAN exposures with depression. However, Egger’s tests (*β* = 2.03, 95% CI: 0.84∼3.21, *P* = 0.044) and funnel plots for the associations of outdoor ALAN exposures and depression suggested the presence of several studies with large effect estimates, potentially influencing the overall pooled results.

## 4. Discussion

Investigating the associations between ALAN and depression would be beneficial for the development of public health strategies aimed at mitigating the adverse effects of ALAN on mental health. Our findings suggest that both outdoor and indoor ALAN exposures are associated with an increased risk of depression. Subgroup analyses for outdoor ALAN indicated that development level, sample size, age group, sex, study design, modality of depression assessment, or adjustment of sleep-related variables in models may not be potential sources of heterogeneity. Sensitivity analyses confirmed the robustness of the findings, while evidence of publication bias was observed for studies on outdoor ALAN.

Our findings indicated that both outdoor and indoor ALAN exposures were positively associated with an increased risk of depression. Specifically, a 1 nW/cm^2^/sr increase in outdoor ALAN was associated with a 0.43% increase in depression risk, which were generally consistent with previous studies [9, 22, 23, 25, 26]. The significant heterogeneity observed among studies on outdoor ALAN exposure suggests that factors such as geographical location, measurement methods, and environmental conditions may influence the observed associations [11, 29]. Nevertheless, our subgroup analyses did not identify development level, sample size, age group, sex, study design, modality of depression assessment, or adjustment of sleep-related variables in models as potential sources of heterogeneity, indicating that other variables might contribute to the differences across studies.

As for indoor ALAN, a 1 lux increase in indoor ALAN was associated with a 3.29% increase in depression risk. The effect estimates were also similar to the two studies included [8, 24]. The lack of significant heterogeneity in studies on indoor ALAN exposure suggests potential consistent effects of indoor ALAN on risk of depression. Nevertheless, considering the limited number (n = 2) and study population (only in Japan) of studies included, more studies are still warranted to examine the robustness and generalizability of current findings. In addition, it is important to note that the participants in the two studies were drawn from the same cohort in Japan, which resulted in some duplicated participants.

The biological mechanisms underlying the associations of ALAN and depression may be attributable to the disruption of circadian rhythms induced by ALAN [32, 33]. Exposure to ALAN is known to suppress melatonin production, a critical hormone for maintaining circadian and sleep regularity [5, 19, 33]. Disruptions in circadian rhythms can adversely affect sleep quality and mood stability, resulting in an increased risk of depression [5, 25]. Meanwhile, the direct impact of ALAN on neurotransmitter systems involved in mood regulation, including serotonin and dopamine pathways, may also be one explanation for our findings [34–36].

Interestingly, only developed regions showed a significant association between outdoor ALAN and depression, while there was no significant difference in the effect estimates between developed and developing regions. Meanwhile, it is noteworthy that we observed greater effect estimates in developing countries compared to developed countries. The lack of statistical significance may be influenced by the relatively small sample size, as the subgroup of developing countries included only two studies. Variability in infrastructure and socioeconomic factors may contribute to the heterogeneity observed in the associations [37, 38]. Meanwhile, outdoor ALAN is an important marker for regional urbanicity [39, 40]. Higher urbanization levels are associated with higher levels of social fragmentation, traffic-related pollution, and reduced access to natural environments [40–42], which could contribute to an increased risk of depression [16–18, 43]. These findings suggest that the associations between outdoor ALAN and depression may be influenced by broader socioeconomic factors in the context of urbanization [41, 43].

In addition, in examining the effects of ALAN on depression, it is crucial to consider the role of natural lighting conditions influenced by geographic latitude. For instance, a meta-analysis reported stronger associations between ALAN and breast cancer in studies conducted in regions with longer sunshine hours [13]. Our meta-analysis primarily included studies from mid-latitude regions in the Northern Hemisphere (the UK, the US, China, Japan, and Korea). Accordingly, further studies including diverse geographic locations, especially varying latitudes, are warranted to provide insights into how natural lighting conditions interact with artificial lighting in affecting mental health.

Our meta-analysis for the first time confirmed the associations of outdoor and indoor ALAN with depression by a systematic review of current publications. Despite the insights provided by this meta-analysis, several limitations should be acknowledged. First, the limited number (5 for outdoor ALAN and 2 for indoor ALAN) and geographical distributions (i.e. China, Japan, Korea, UK, and USA) of the included studies limited the generalizability of our findings. Meanwhile, the limited number of studies (2 cohort studies and 3 cross-sectional studies) also constrained further dose-response meta-analysis for outdoor ALAN exposure. Second, most studies were cross-sectional studies, and this study design may preclude the establishment of causality. Further prospective studies are thus needed to verify the findings on associations of ALAN and depression. Third, the presence of publication bias for studies on outdoor ALAN suggests that the pooled associations might be influenced by the selective reporting of studies with positive results. However, it should be noted that the limited number of included studies may restrict the interpretability of the results from the Begg’s and Egger’s tests. Fourth, previous studies did not investigate the mediating effects of sleep in the association between ALAN and depression. Further studies are thus warranted to evaluate the mediating role of sleep in the association between ALAN and depression, in order to provide a more comprehensive understanding of the mental health effects of ALAN.

## 5. Conclusion

In conclusion, our systematic review and meta-analysis demonstrated that both outdoor and indoor ALAN exposures are associated with an increased risk of depression. These findings underscore the importance of considering ALAN as a significant environmental risk factor for mental disorders. These results advocate for the integration of light pollution mitigation strategies, particularly for regions and individuals with stronger exposures to ALAN. Further research with prospective and experimental designs is warranted to confirm our findings and to better understand the underlying mechanisms.

## Supplementary information

**Additional file 1:** **Table S1.** Details of search strategy used in the five literature databases. **Table S2.** The detailed criteria of the Newcastle-Ottawa Scale (NOS). **Table S3.** Quality of the included study according to the Newcastle-Ottawa Scale (NOS). **Table S4.** Results of sensitivity analyses omitting one study each at a time for outdoor ALAN. **Table S5.** Results of Begg’s test and Egger’s test. **Figure S1.** Funnel plots of publication bias for the association between outdoor ALAN and depression.

## Declarations

### Availability of data and material

All data used in this study are publicly available.
