# Supplementary material for: Artificial light at night and risk of depression: a systematic review and meta-analysis
Source: Environ Health Prev Med. 2024 Dec 26;29:73. doi: 10.1265/ehpm.24-00257 (PMC11701095; doi:10.1265/ehpm.24-00257)
Supplement: Supplementary file 1 — Additional file 1: Table S1. Details of search strategy used in the five literature databases. Table S2. The detailed criteria of the Newcastle-Ottawa Scale (NOS). Table S3. Quality of the included study according to the Newcastle-Ottawa Scale (NOS). Table S4. Results of sensitivity analyses omitting one study each at a time for outdoor ALAN. Table S5. Results of Begg’s test and Egger’s test. Figure S1. Funnel plots of publication bias for the association between outdoor ALAN and depression. [file ehpm-29-073-s001.docx]

**Supplementary materials**

**Artificial light at night and risk of depression: A systematic review and meta-analysis**

**Authors:** Manman Chen^1*^, Yuankai Zhao^1*^, Qu Lu^1^, Zichen Ye^1^, Anying Bai^1^, Zhilan Xie^1^, Daqian Zhang^1^, Yu Jiang^2,1#^

**Affiliations:**

^1^ School of Population Medicine and Public Health, Chinese Academy of Medical Sciences and Peking Union Medical College, Beijing, China;

^2^ School of Health Policy and Management, Chinese Academy of Medical Sciences and Peking Union Medical College, Beijing, China;

***Co-first author:** M.C. and Y.Z. contributed equally to this work.

**#Correspondence to：**Yu Jiang, School of Health Policy and Management, Chinese Academy of Medical Sciences and Peking Union Medical College, Beijing, China; E-mail: jiangyu@pumc.edu.cn

Table S1. Details of search strategy used in the five literature databases

| Literature database | Search strategy |
| --- | --- |
| PubMed | #1 "Light Pollution"[MeSH Terms] OR "Light Pollution"[Title/Abstract] OR "light exposure"[Title/Abstract] OR "artificial light"[Title/Abstract] OR "night light"[Title/Abstract] OR "bedroom light"[Title/Abstract] OR "light at night"[Title/Abstract] OR "environmental light*"[Title/Abstract] OR "ambient light"[Title/Abstract] OR "domestic light"[Title/Abstract]  #2 "Depression"[MeSH Terms] OR "Depressive Disorder"[MeSH Terms] OR "Suicide"[MeSH Terms] OR "Depression"[Title/Abstract] OR "Depressive Disorder"[Title/Abstract] OR "depressive symptom*"[Title/Abstract] OR "depressed mood"[Title/Abstract] OR "Suicide"[Title/Abstract]  #1 AND #2  ("Light Pollution"[MeSH Terms] OR ("Light Pollution"[Title/Abstract] OR "light exposure"[Title/Abstract] OR "artificial light"[Title/Abstract] OR "night light"[Title/Abstract] OR "bedroom light"[Title/Abstract] OR "light at night"[Title/Abstract] OR "environmental light*"[Title/Abstract] OR "ambient light"[Title/Abstract] OR "domestic light"[Title/Abstract])) AND ("Depression"[MeSH Terms] OR "Depressive Disorder"[MeSH Terms] OR "Suicide"[MeSH Terms] OR ("Depression"[Title/Abstract] OR "Depressive Disorder"[Title/Abstract] OR "depressive symptom*"[Title/Abstract] OR "depressed mood"[Title/Abstract] OR "Suicide"[Title/Abstract])) |
| Web of Science | (TS=("light pollution" OR "light exposure" OR "artificial light" OR "night* light" OR "bedroom light" OR "light at night" OR "environmental light*" OR "ambient light" OR "domestic light")) AND TS=("depression" OR "depressive disorder" OR "depressive symptom*" OR "depressed mood" OR "suicide") |
| EMBASE | #1 'light pollution'/exp OR 'light pollution':ti,ab,kw OR 'light exposure':ti,ab,kw OR 'artificial light':ti,ab,kw OR 'night* light':ti,ab,kw OR 'bedroom light':ti,ab,kw OR 'light at night':ti,ab,kw OR 'environmental light*':ti,ab,kw OR 'ambient light':ti,ab,kw OR 'domestic light':ti,ab,kw  #2 'depression'/exp OR 'suicide'/exp OR 'depression':ti,ab,kw OR 'depressive disorder':ti,ab,kw OR 'depressive symptom*':ti,ab,kw OR 'depressed mood':ti,ab,kw OR 'depressed mood':ti,ab,kw OR 'suicide':ti,ab,kw  #3 #1 AND #2 |
| Cochrane | #1 MeSH descriptor: [Light Pollution] explode all trees 1  #2 (light pollution):ti,ab,kw OR (light exposure):ti,ab,kw OR (artificial light):ti,ab,kw OR (night* light):ti,ab,kw OR (bedroom light):ti,ab,kw OR (light at night):ti,ab,kw OR (environmental light*):ti,ab,kw OR (ambient light):ti,ab,kw OR (domestic light):ti,ab,kw  #3 #1 OR #2  #4 MeSH descriptor: [Depression] explode all trees  #5 MeSH descriptor: [Suicide] explode all trees  #6 (depression):ti,ab,kw OR (depressive disorder):ti,ab,kw OR (depressive symptom):ti,ab,kw OR (depressed mood):ti,ab,kw OR (suicide):ti,ab,kw  #7 #4 OR #5 OR #6  #8 #3 AND #7 |
| Ovid | ((light pollution or light exposure or artificial light or night* light or bedroom light or light at night or environmental light* or ambient light or domestic light) and (depression or depressive symptom* or depressive disorder or depressed mood or suicide)).ti,ab,kw. |

Table S2. The detailed criteria of the Newcastle-Ottawa Scale (NOS)

**Quality assessment tool of cohort studies**

Newcastle-Ottawa Scale

*Selection: (Maximum 4 stars)*

1) Representativeness of the exposed cohort:

a) Truly representative of the average in the target population. * (all subjects or random sampling)

b) Somewhat representative of the average in the target population. * (non-random sampling)

c) Selected group of users.

d) No description of the sampling strategy.

1. Selection of the non exposed cohort:
2. Drawn from the same community as the exposed cohort. *
3. Drawn from a different source.
4. No description of the derivation of the non exposed cohort.
5. Ascertainment of exposure:
6. Secure record (eg surgical records). *
7. Structured interview. *
8. Written self report.
9. No description.
10. Demonstration that outcome of interest was not present at start of study:
11. Yes. *
12. No.

*Comparability: (Maximum 2 stars)*

1) The subjects in different outcome groups are comparable, based on the study design or analysis. Confounding factors are controlled.

a) The study controls for the most important factor (particulate matter air pollution, which has been associated with depression ^[1]^, and may have interactive effects with ALAN on health ^[2]^). *

b) The study control for additional factors including demographic variables (sex, age, living regions, income, education, etc.) and medical history (chronic disease diagnoses, medications, etc.). *

*Outcome: (Maximum 3 stars)*

1) Assessment of the outcome:

a) Independent blind assessment. **

b) Record linkage. **

c) Self report. *

d) No description.

1. Was follow-up long enough for outcomes to occur:
2. Yes. (5 yrs. for exposure to depression) *
3. No.
4. Adequacy of follow up of cohorts:
5. Complete follow up -all subjects accounted for. *
6. Subjects lost to follow up unlikely to introduce bias - small number lost - > 90% follow up, or description provided of those lost) *
7. Follow up rate < 90 % and no description of those lost
8. No statement

**Quality assessment tool of cross-sectional studies**

Newcastle-Ottawa Scale adapted for cross-sectional studies

*Selection: (Maximum 5 stars)*

1) Representativeness of the sample:

a) Truly representative of the average in the target population. * (all subjects or random sampling)

b) Somewhat representative of the average in the target population. * (non-random sampling)

c) Selected group of users.

d) No description of the sampling strategy.

2) Sample size:

a) Justified and satisfactory. *

b) Not justified.

3) Non-respondents:

a) Comparability between respondents and non-respondents characteristics is established, and the response rate is satisfactory. *

b) The response rate is unsatisfactory, or the comparability between respondents and non-respondents is unsatisfactory.

c) No description of the response rate or the characteristics of the responders and the non-responders.

4) Ascertainment of the exposure (risk factor):

a) Validated measurement tool. **

b) Non-validated measurement tool, but the tool is available or described.*

c) No description of the measurement tool.

*Comparability: (Maximum 2 stars)*

1) The subjects in different outcome groups are comparable, based on the study design or analysis. Confounding factors are controlled.

a) The study controls for the most important factor (particulate matter air pollution, which has been associated with depression ^[1]^, and may have interactive effects with ALAN on health ^[2]^). *

b) The study control for additional factors including demographic variables (sex, age, living regions, income, education, etc.) and medical history (chronic disease diagnoses, medications, etc.). *

*Outcome: (Maximum 3 stars)*

1) Assessment of the outcome:

a) Independent blind assessment. **

b) Record linkage. **

c) Self report. *

d) No description.

2) Statistical test:

a) The statistical test used to analyze the data is clearly described and appropriate, and the measurement of the association is presented, including confidence intervals and the probability level (p value). *

b) The statistical test is not appropriate, not described or incomplete.

Table S3. Quality of the included study according to the Newcastle-Ottawa Scale (NOS)

A/Quality assessment of cohort studies included in this review based on the NOS (N=3)

Note: Scores of 7–9, 4–6, and 4 were classified as having a low (high quality), moderate (moderate quality), or high (low quality) risk of bias, respectively.

| **Study Name** | **Selection** | | | | **Comparability** | **Outcome** | | | **Total**  **(9⋆)** |
| --- | --- | --- | --- | --- | --- | --- | --- | --- | --- |
|  | Representative-ness of  exposed cohort (⋆) | Selection of  non-exposed  cohort (⋆) | Ascertainment of  exposure (⋆) | Demonstration that outcome of interest was not present at start of study (⋆) | Comparability (⋆⋆) | Assessment of  outcome (⋆) | Follow-up long enough for outcomes to occur  (⋆) | Adequacy of follow-up of cohorts  (⋆) |  |
| Jin et al (2023) | * | * | * | * | ** | * | * | * | 9 |
| Yu et al (2022) | * | * | - | - | * | * | * | * | 6 |
| Obayashi et al (2018) | * | * | * | * | * | * | * | - | 7 |

| **Study Name** | **Selection** | | | | **Comparability** | **Outcome** | | **Total**  **(10⋆)** |
| --- | --- | --- | --- | --- | --- | --- | --- | --- |
|  | Representativeness of the sample (⋆) | Sample size (⋆) | Non-respondents (⋆) | Ascertainment of the exposure (risk factor) (⋆⋆) | Comparability (⋆⋆) | Assessment of outcome (⋆⋆) | Statistical test (⋆) |  |
| Paksarian et al (2020) | * | * | - | ** | * | ** | * | 8 |
| Obayashi et al (2022) | * | * | * | ** | * | * | * | 8 |
| Zhu et al (2023） | - | * | - | ** | * | * | * | 6 |
| Min et al (2018） | * | * | - | ** | ** | * | * | 8 |

B/Quality assessment of cross-sectional studies included in the systematic review based on the NOS (N=4)

Note: Scores of 7–9, 4–6, and 4 were classified as having a low (high quality), moderate (moderate quality), or high (low quality) risk of bias, respectively.

Table S4. Results of sensitivity analyses omitting one study each at a time for outdoor ALAN

| Article | Estimate | *P*-value |
| --- | --- | --- |
| Omitting Jin et al (2023) | 0.47% (0.29%~0.65%) | <0.001 |
| Omitting Min et al (2018) | 0.36% (0.17%~0.55%) | <0.001 |
| Omitting Paksarian et al (2020) | 0.42% (0.25%~0.59%) | <0.001 |
| Omitting Yu et al (2022) | 0.42% (0.26%~0.57%) | <0.001 |
| Omitting Zhu et al (2023) | 0.45% (0.26%~0.64%) | <0.001 |
|  |  |  |
| Pooled estimate | 0.43% (0.21%~0.65%) | <0.001 |

Note: Estimates were shown as pooled percent changes (%) and 95% confidence intervals (CIs) in risk of depression associated with 1 nW/cm^2^/sr increase in outdoor ALAN.

Table S5. Results of Begg’s test and Egger’s test

| Exposure | Study number | Coefficient of Begg’s test | *P*-value of Begg’s test | Coefficient of Egger’s test | *P*-value of Egger’s test |
| --- | --- | --- | --- | --- | --- |
| Outdoor ALAN | 5 | 2.00 (95% CI: -6.00, 10.00) | 0.624 | 2.03 (95% CI: 0.84, 3.21) | 0.044 |
| Indoor ALAN | 2 | — | — | — | — |

Note: The tests were not calculated for studies on indoor ALAN due to the limited study number (n = 2).


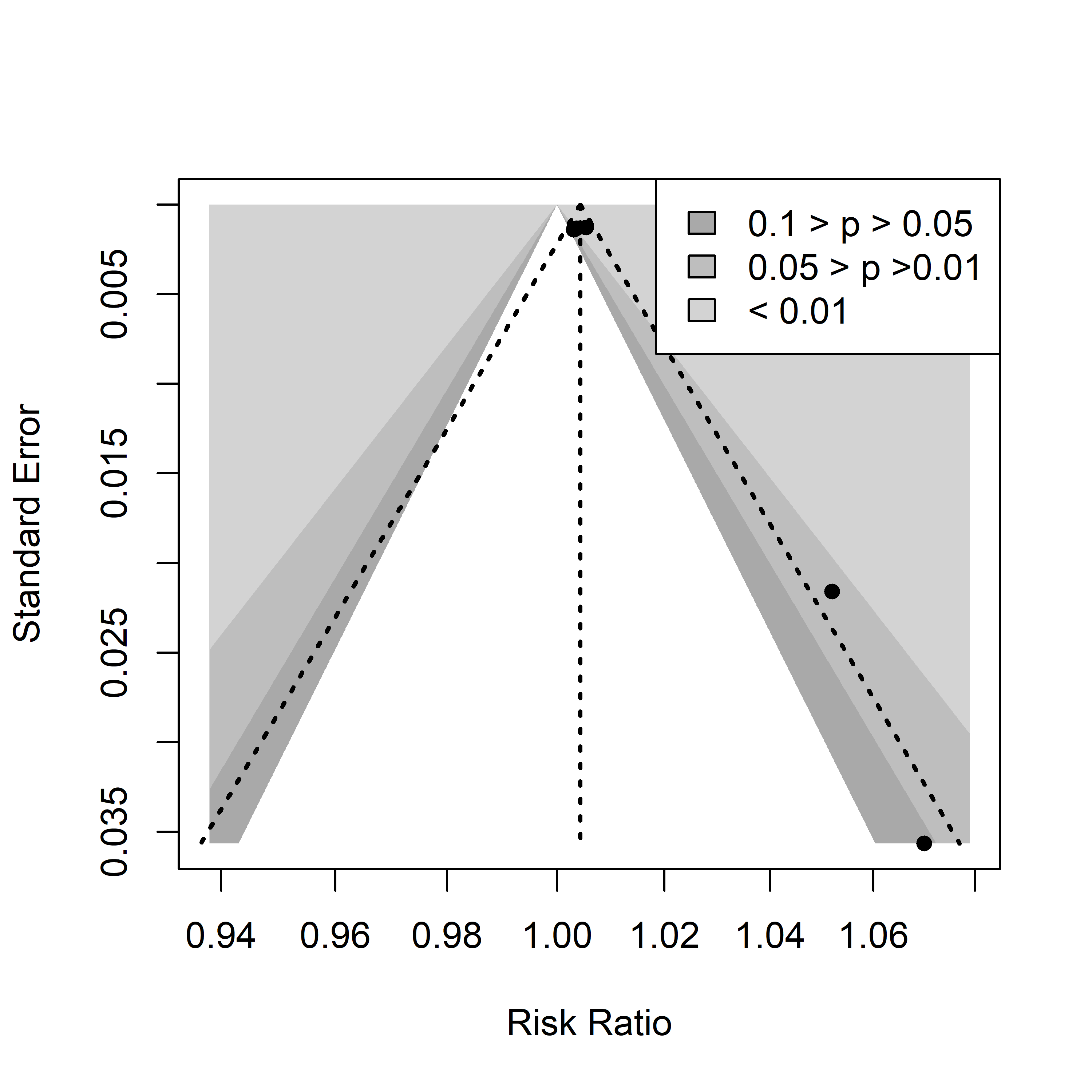


Figure S1. Funnel plots of publication bias for the association between outdoor ALAN and depression

References:

1. Borroni, E, Pesatori, AC, Bollati, V, et al. Air pollution exposure and depression: A comprehensive updated systematic review and meta-analysis [J]. Environ Pollut, 2022, 292(Pt A): 118245.

2. Liang, Z, Wang, W, Wang, Y, et al. Urbanization, ambient air pollution, and prevalence of chronic kidney disease: A nationwide cross-sectional study [J]. Environ Int, 2021, 156: 106752.
